# Assessing Student and Physician Fertility Awareness Utilizing the Fertility and Infertility Treatment Knowledge Score (FIT-KS)

**DOI:** 10.7759/cureus.70244

**Published:** 2024-09-26

**Authors:** Martha Doxsey, Krishna Patel, Kristin Faschan, Lilia Reyes

**Affiliations:** 1 Obstetrics and Gynecology, Tufts Medical Center, Boston, USA; 2 Medical Education, Penn State University College of Medicine, Milton S. Hershey Medical Center, Hershey, USA; 3 Emergency Medicine, WellSpan Good Samaritan Hospital, Lebanon, USA; 4 Pediatric Emergency Medicine, Penn State University College of Medicine, Milton S. Hershey Medical Center, Hershey, USA

**Keywords:** family planning, female physicians, medical education curriculum, medical resident education, obstetrics and gynecology (ob-gyn), obstetrics and gynecology residency, pregnancy and family planning

## Abstract

Introduction: Medical trainees are delaying childbearing due to competing demands of career, financial constraints, and limited parental leave. Delaying childbearing increases the risk of obstetric complications, with up to one-quarter of female physicians facing infertility. Education on family planning and fertility is rarely discussed among medical trainees, and research on medical trainees' knowledge of fertility and infertility is minimal.

Methods: Subjects included medical students, residents, fellows, and attendings assigned female at birth. The distributed survey included the Fertility and Infertility Treatment Knowledge Score (FIT-KS) instrument to assess for fertility and infertility knowledge. Outcome measures include participant age, stage of training, parity, specialty, and FIT-KS.

Results: Of the 291 participants included in the final analysis, participants scored an average of 20/29 (69%) on the FIT-KS. Around 44.8% (n=130) of participants overestimated the fecundability of a 30-year-old woman, and 36.9% (n=107) underestimated the chance of miscarriage in peak reproductive years. Five out of eight questions pertaining to infertility treatment had a <50% correct response rate. About 76.6% (n=216) of participants underestimated in vitro fertilization (IVF) success for a woman <35 years old, and 56% (n=158) overestimated the success of IVF for a woman >44 years old. Around 43.6% (n=123) of participants overestimated the average cost of IVF. Of the main outcome measures, only those participants representing obstetrics and gynecology (OB-GYN) performed better on average than all other specialties (FIT-KS=21.7 vs. 19.5).

Conclusion: Participants overestimated fecundity with increasing age and had insufficient knowledge of infertility treatment success. More awareness and early discussion about family planning and fertility goals are needed in medical training.

## Introduction

According to the most recent US Census data, from 1990 to 2019, birth rates for women aged 20-24 declined by 42.8% and increased 67.4% for women aged 35-39 [1]. Following this trend, the median age of first birth increased from 24.6 years in 2000 to 26.7 years in 2016 [2].

There are a wide range of reasons as to why women delay childbearing. For medical students and physicians, the conflict between career and pregnancy in the peak reproductive years is a commonly cited reason, with 83% of female physicians delaying pregnancy due to competing career demands [3-7]. On average, physicians complete medical training at age 31 years, and the age when most female physicians give birth is 32 years [8], delayed from their nonphysician counterparts at 26 years [2] and women with advanced degrees at 30 years [9]. Only 2-10% of female physicians reported having a pregnancy during medical school [8,10], and 30-40% of female residents have children during residency training [10,11]. Family planning decisions are most affected by physicians' busy and unmodifiable work schedules, limited maternity leave, financial constraints, decision to pursue further specialized training, lack of childcare, lactation concerns, burden on colleagues, concern for maternal-fetal wellbeing, and desire to avoid training extensions [4,7,10,12-16].

Increased risk of obstetric complications in residency, including the risk of preterm labor and delivery, preeclampsia, gestational diabetes, hypertension, placental abruption, fetal growth restriction, low birth weight, and stillbirth, has been well documented in the literature for over two decades [5,10-12,17]. Beyond obstetrical complications, up to one-quarter of female physicians face infertility [6,10]. A survey of female physicians who graduated from medical school between 1995 and 2000 found that 21% took longer than one year to conceive their first pregnancy, which is twice the national ﻿average, and 7% were unable to ever become pregnant [6]. One study found that among female physicians reporting difficulty with fertility, 84% underwent an infertility evaluation and 76% utilized assisted reproductive technology (ART) [18]. Despite the high rate of female physician infertility, there is insufficient data showing how much female physicians know about their fertility and the potential consequences of delaying childbearing.

Family planning and female physician infertility are rarely discussed in medical school, residency, or subsequent practice. One study found that more than half (53.3%) of female physicians would have attempted to conceive earlier if they had known infertility would be an issue [6]. To date, there have been very few projects on medical trainees' knowledge of fertility across the training spectrum. Therefore, we used the validated 29-item Fertility and Infertility Treatment Knowledge Score (FIT-KS) instrument developed by Kudesia et al. to analyze fertility and infertility knowledge among medical students, residents, fellows, and attending physicians assigned female at birth [19].

## Materials and methods

Study design

Permission was obtained to use the FIT-KS, developed by Kudesia et al. [19]. The FIT-KS is a survey with 29 multiple-choice items assessing natural fertility (21 questions) and infertility treatment (eight questions) knowledge. It has previously been validated in the United States with reproductive-aged women as well as in female medical trainees [19]. Relevant items on the FIT-KS instrument were reviewed against the updated Society for Assisted Reproductive Technology (SART) and 2019 Centers for Disease Control and Prevention (CDC) [20] data for accuracy. Discrepancies between the FIT-KS and the 2019 CDC and SART data were resolved in discussion with a board-certified reproductive endocrinology and infertility subspecialist. Item 25 was corrected to reflect the trends in in vitro fertilization (IVF) towards single-embryo transfers and therefore fewer twin pregnancies (closer to 6% nationally). All other items of the FIT-KS remained accurate. Of note, the 2019 CDC and SART data utilized the language of "live birth rate" instead of "pregnancy rate" on the original survey for items 23 and 24. We did not alter the language of the survey to avoid impacting the validity, as we determined the answer remained correct regardless of phrasing. The FIT-KS instrument with updated items can be found in Appendix A and Appendix B.

Survey dissemination and eligible participants

The FIT-KS was uploaded into REDCap. In April of 2022, all medical students, residents, fellows, and attending physicians at Penn State College of Medicine and Milton S. Hershey Medical Center received an email describing the study and a link to complete the survey. We chose to include participants who are cis-gender women, assigned female at birth due to the documented impact of female physician infertility present in the literature currently. A follow-up email was sent in July of 2022 to prompt further responses. All responses were anonymous. Participation was voluntary and uncompensated, and completion of the survey was considered implied consent. Our study was found to be exempt and approved by the Penn State University Institutional Review Board (approval number: STUDY00019216). Demographic information pertaining to age, parity, previous ART use, marital status, race, ethnicity, religion, income, area of geographical upbringing, level of training, and specialty was collected.

Statistics

Descriptive statistics, including means, medians, standard deviations, and confidence intervals, were generated for continuous variables; frequency tables were calculated for categorical variables. The 95% confidence intervals reflect 0.025 in each tail or p-values no higher than 0.05. The statistical analysis was performed using SAS software, version 9.4. 

## Results

Demographics

Three hundred eleven participants responded to the survey. Twenty participants only completed demographic information and were excluded from the analysis, leaving 291 participants. Five participants did not answer demographic information but did respond to the FIT-KS survey and were included in the main analysis. Participants were included if any of the FIT-KS instrument was completed.

As outlined in Table 1 (n=286), the ages of participants ranged from 22 to 72 years old with a mean age and standard deviation of approximately 34±10.7 years old and a median of 30 years. A majority of participants were married (52.1%, n=149), White (71.3%, n=204), and Christian (55.9%, n=160). Around 48.3% (n=138) of participants were raised in the Mid-Atlantic region. There was representation across the spectrum of medical training and specialties.

**Table 1:** Descriptive characteristics and demographics of participants (n=286)

Descriptive characteristics and demographics	Frequency (n)	Percentage (%)
Marital status		
Single	60	20.98
In a relationship	68	23.78
Married	149	52.10
Divorced/separated	9	3.15
Race		
Black	5	1.75
White	204	71.33
Asian	51	17.83
Multiracial	9	3.15
Other	7	2.45
Ethnicity		
Hispanic	10	3.50
Non-Hispanic	276	96.50
Religion		
Christianity	160	55.94
Judaism	9	3.15
Muslim	8	2.80
Agnostic	38	13.29
Atheist	40	13.99
Other	31	10.84
Income		
<50k	83	29.02
50-100k	105	36.71
100-200k	24	8.39
200-300k	50	17.48
400-500k	24	8.39
Region raised		
New England	23	8.04
Mid-Atlantic	138	48.25
North East Central	54	18.88
West North Central	5	1.75
South Atlantic	12	4.20
East South Central	6	2.10
West South Central	3	1.05
Mountain	5	1.7
Pacific	13	4.55
US Territories	6	2.10
International	21	7.34
Career phase		
MS1	7	2.45
MS2	26	9.09
MS3	22	7.69
MS4	29	10.14
PGY1	41	14.34
PGY2	17	5.94
PGY3	20	6.99
PGY4	19	6.64
PGY5	8	2.80
PGY6	5	1.75
PGY7	2	0.70
Board-certified/board-eligible attending physician	90	31.47
Specialty		
Dermatology	12	4.20
Emergency medicine	16	5.59
Family medicine	17	5.94
Internal medicine	58	20.28
Obstetrics and gynecology	21	7.34
Pediatrics	35	12.24
Radiology	7	2.45
Surgery	22	7.69
Pathology	10	3.50
Anesthesia	21	7.34
Neurology	9	3.15
Ophthalmology	1	0.35
Physical medicine and rehabilitation	8	2.80
Undecided	13	4.55
Other	36	12.59

Most participants (65.4%, n=187) had not given birth to children but desired to (82.4%, n=154). Of those who had previously given birth (n=99), 15% (n=15) had utilized ART. This can be found in Table 2.

**Table 2:** Descriptive characteristics of parity (n=286) IVF: in vitro fertilization; IUI: intrauterine insemination

Parity	Frequency (n)	Percentage (%)
Given birth to children		
Yes	99	34.6
No	187	65.4
Used IVF or IUI		
Yes	15	15.5
No	82	84.5
Desire to give birth		
Yes	154	82.4
No	33	17.7

FIT-KS responses

On average, participants correctly answered 20 of the 29-item FIT-KS instrument, with ranges from three to 27 correct answers. There were nine items that had a <50% correct response rate, four pertaining to fertility knowledge and five pertaining to infertility treatment knowledge (Table 3).

**Table 3:** FIT-KS items with <50% overall correct response rate The asterisks (*) were added to indicate which data in the table has a statistically significant p-value. FIT-KS: Fertility and Infertility Treatment Knowledge Score; OB-GYN: obstetrics and gynecology; IVF: in vitro fertilization; T/F: true/false

FIT-KS	Correct (%)	Incorrect (%)
Fertility knowledge		
Over the course of one month, what is the percent chance that a 30-year-old woman who is trying to get pregnant will get pregnant?	117 (40.3)	173 (59.7) *130 (44.8) overestimated*
	No difference by stage of training (p=0.41)	
	30-39-year-olds answered correctly (p=0.0252)*	
	No difference by specialty (p=0.85)	
	No difference by parity (p=0.65)	
On average, for a woman in her peak reproductive years, what is the percent chance that a pregnancy will end in a miscarriage?	143 (49.3)	147 (50.7) *107 (36.9) underestimated*
	No difference by stage of training (p=0.14)	
	No difference by age (p=0.53)	
	OB-GYN answered correctly (p=0.003)*	
	No difference by parity (p=0.71)	
What is the average survival time of normal sperm in the female reproductive tract?	108 (37.2)	182 (62.8)
	No difference by stage of training (p=0.81)	
	No difference by age (p=0.34)	
	No difference by specialty (p=0.27)	
	No difference by parity (p=0.40)	
Lifestyle and fertility knowledge		
T/F: moderate alcohol consumption affects fertility	125 (43.1)	165 (59.9)
	No difference by stage of training (p=0.09)	
	No difference by age (p=0.17)	
	No difference by specialty (p=0.08)	
	No difference by parity (p=0.24)	
Infertility treatment knowledge		
For a woman <35 years old undergoing IVF with her own eggs, what is the pregnancy rate per cycle?	58 (20.6)	224 (79.4) *216 (76.6) underestimated*
	No difference by stage of training (p=0.80)	
	No difference by age (p=0.29)	
	No difference by specialty (p=0.64)	
	No difference by parity (p=0.46)	
For a woman over 44 years old, undergoing IVF with her own eggs, what is the pregnancy rate per cycle?	124 (44)	158 (56) *158 (56) overestimated*
	No difference by stage of training (p=0.85)	
	No difference by age (p=0.48)	
	No difference by specialty (p=0.093)	
	No difference by parity (p=0.41)	
In women undergoing IVF, what is the percentage of pregnancies that result in twins?	124 (44)	158 (56) *120 (42.6) overestimated*
	Medical student answered incorrectly (p=0.0057)*	
	<25-year-olds answered incorrectly (p=0.0054)*	
	No difference by specialty (p=0.88)	
	Parous answered correctly (p=0.0003)*	
Average cost of an IVF cycle in the United States	66 (23.4)	216 (76.6) *123 (43.6) overestimated*
	No difference by stage of training (p=0.37)	
	No difference by age (p=0.099)	
	No difference by specialty (p=0.60)	
	No difference by parity (p=0.17)	
When using frozen eggs from women <37 years old, what is the live birth rate per thawed egg?	41 (14.5)	241 (85.5) *241 (85.5) overestimated*
	Medical students answered incorrectly (p=0.045)*	
	No difference by age (p=0.061)	
	No difference by specialty (p=0.48)	
	Parous answered correctly (p=0.0039)*	

In reviewing individual FIT-KS items, more participants incorrectly answered the percent chance a 30-year-old woman who is trying to get pregnant will do so over the course of one month, with 44.8% (n=130) of those who answered incorrectly overestimating the percent chance. Of note, those participants aged 30-39 more often identified the percent chance that a 30-year-old woman who is trying to get pregnant will get pregnant over the course of one month (p=0.025). Approximately half of the participants incorrectly answered the percent chance a pregnancy will end in a miscarriage during a woman's peak reproductive years. Those in obstetrics and gynecology (OB-GYN) answered this correctly (p=0.003). Those >40 years old, attendings, and parous participants more often identified the optimal time to have sexual intercourse to achieve pregnancy (p=0.048, p=0.006, p=0.014) and that using certain types of sexual lubricants affects fertility (p=0.015, p=0.001, p=0.024). However, participants >40 years old had difficulty identifying that prior use of oral contraceptive pills did not affect fertility (p=0.0201). Medical students more often correctly identified where fertilization most commonly occurs (p=0.015), while those aged 30-39 and parous patients correctly identified this least often (p=0.003, p=0.048). Only 20.6% (n=58) of participants were able to correctly identify the pregnancy rate per cycle for women <35 years old undergoing IVF. Around 79.4% (n=224) answered this incorrectly with 76.6% (n=216) of those answering incorrectly underestimating the pregnancy rate per cycle. About 76.6% (n=216) of participants also incorrectly identified the average cost of an IVF cycle in the United States; 43.6% (n=126) of individuals overestimated this value. Both of these items did not differ across age, stage of training, specialty, or parity. Parous participants more often correctly identified the percentage of pregnancies that result in twins in a woman undergoing IVF (p<0.001) and the live birth rate per thawed egg when using frozen eggs from women <37 years old (p=0.004), while medical students were least likely to correctly answer these items (p=0.006, p=0.0466). These findings are highlighted above in Table 3.

## Discussion

Our study assessed fertility and infertility treatment knowledge among medical students and physicians assigned female at birth. Previous research has led to the assessment of "fertility awareness" as a concept defined as "the understanding of reproduction, fecundity, fecundability, and related individual and non-individual risk factors, including the awareness of societal and cultural factors affecting options to meet reproductive family planning, as well as family building needs" [21,22]. Fertility knowledge among the general public is low, with one study finding that the mean score on the FIT-KS was 16.2±3.5 (55.9% correct) among the general population [19,21]. However, fertility awareness has also been documented to be low among undergraduate students [23], medical trainees [19,24], and OB-GYN residents, indicating a need for continued and/or enhanced education in fertility throughout training [25]. Common misperceptions reported in these studies included the age at which fertility declines [23,24], an overestimation of ART success [19,23,26-29], and awareness of oocyte cryopreservation [24]. This evidence coincides with our study's results that found medical trainees to score an average of 20 correct on the 29-item FIT-KS survey, with a <50% correct response rate on nine items. Although the average score on the FIT-KS survey is higher among medical trainees when compared to the general population, the lower scores on the survey, particularly on items on infertility knowledge, raise the question of a need to improve education on fertility and infertility for medical trainees.

Our questionnaire found that participants tended to perform better on the fertility knowledge portion of the FIT-KS instrument than on the infertility treatment knowledge portion. In concordance with previous studies, challenging questions addressed declines in fecundity with age and IVF success statistics. Participants generally recognized that fecundity decreases with age but overestimated successful pregnancy rates with increasing age and underestimated miscarriage rates. This is important as we know medical trainees tend to delay childbearing [3-7].

Studies have also described "the motherhood penalty" and highlighted the need for the Accreditation Council for Graduate Medical Education (ACGME) reform to include family planning options for resident physicians [13]. Although there is some education on fertility in medical school, the current education that exists for both medical students and resident physicians lacks additional educational opportunities focused on infertility and family planning. Previous studies found that having this education would prospectively change family planning decisions, showing that 53.3% of female physicians would have attempted to conceive earlier if they had known infertility would be an issue [6]. Our findings corroborate the need for enhanced education regarding fertility as well as increased conversation about, and institutional support for, optimal family planning timing within a medical career. These educational opportunities should be available for all medical trainees, regardless of sex and gender identity.

Fertility education should be presented both during medical school and in residency training as early and continued education on the subject will facilitate family planning [6]. Steps towards implementation should be focused on expanding our current medical curriculum to include topics on infertility and family planning. Education on access to fertility preservation services should also be provided to medical trainees who are interested in family planning at any point in their careers. Supporting physicians balancing family and career however should go beyond education. A previous study found that many medical schools do not currently provide parental leave. However, a mandate from the ACGME states that institutions must have policies that include six weeks or more of paid parental leave, an important step in family planning reform. It is essential for these policies to also include parental leave for both birth and nonbirth parents to counteract the burden that often falls on women for childcare responsibilities. For these steps to be implemented, buy-in at the institutional level is needed. Institutional buy-in can be gained by proposing paid leave as a tool for both retaining medical trainees and enhancing the appeal of their medical program to attending physicians and medical trainees.

The limitations of our study include an inability to accurately calculate a response rate as an open survey was sent to all medical students, residents, fellows, and attendings at our institution. Additionally, while our sample represents the demographics of a single-center institution, there was a predominance of married White Christian women raised in the Mid-Atlantic region. Surveys conducted of a more diverse institution or with multiple institutions would enhance the generalizability of our findings. We also chose to survey participants who are cis-gender women, assigned female at birth due to the documented impact of female physician infertility in the literature. However, we acknowledge that family-building decisions and conversations surrounding fertility are different for individuals and couples of all gender identities. Knowing how these individuals and their partners view fertility issues is an important piece of this decision-making process. Investigating how fertility education also differs for people who can become pregnant who are not cis-gender women (e.g., non-binary and/or transgender men assigned female at birth) is also an important and much-needed area of research [30]. Exploring this difference in knowledge will also help in addressing inequities in access to care and in the medical school curriculum.

Despite these limitations, our findings model those of previous studies and indicate the need for enhanced education and discussion surrounding family planning in medical training. Two-thirds of our participants were under 40 years of age, which may explain the large portion who had not yet given birth to children but desired them. This provides us with an opportunity for future engagement with participants. We could further elucidate the reasoning behind delays in childbearing through focus groups to better determine when discussions around family planning would be most beneficial (e.g., medical school vs. residency) as well as how to increase the transparency of institutional policies in place to support medical students and residents in their family planning decisions.

## Conclusions

With the previously documented high rates of infertility among female medical trainees and physicians, it is of concern that participants overestimated fecundity with increasing age and had insufficient knowledge of infertility treatment success. More awareness and early discussion about family planning and fertility goals are needed in medical training, with an additional focus on policy reform for family planning.

## Appendices

Appendix A

FIT-KS Instrument

The following questions relate to natural fertility:

1) At what age are women most fertile?             

            □ 12-19           

            □ 20-29*                     

            □ 30-39           

            □ 40-49           

2) Over which age range does a woman's ability to get pregnant decline most precipitously?        

            □ 25-29           

            □ 30-34           

            □ 35-39*                     

            □ 40-45           

3) Over the course of one month, what is the percent chance that a 30-year-old woman who is trying to get pregnant will get pregnant?       

            □ 10%             

            □ 20%*           

            □ 30%             

            □ 40%             

4) Over the course of one month, what is the percent chance that a 40-year-old woman who is trying to get pregnant will get pregnant?

            □ ≤5%*           

            □ 6-10%                      

            □ 11-15%        

            □ 16-20%        

5) On average, for a woman in her peak reproductive years, what is the percent chance that a pregnancy (recognized or unrecognized) will end in a miscarriage?

            □ ≤5%             

            □ 6-15%                      

            □ 16-25%*      

            □ 26-35%        

6) A woman and a man can both contribute to a couple's infertility:

            □ True*           

            □ False            

7) A man's age is a factor that affects a couple's fertility:                       

            □ True*           

            □ False            

8) Having less than nine periods in a year can be normal for some women and doesn't require any further evaluation:

           □ True             

           □ False*                                              

9) What is the average survival time of normal sperm in the female reproductive tract?

           □ 12-24 hours  

           □ 24-48 hours  

           □ 3-5 days*     

           □ 6-9 days                                                       

10) When is the optimal time to have sexual intercourse in order to get pregnant?

            □ Right before the period starts             

            □ First few days of the period               

            □ About halfway through the cycle*      

            □ It doesn't matter                                

11) Where does fertilization most commonly occur?

            □ In the uterus                          

            □ Inside the ovaries                  

            □ On the surface of the ovaries  

            □ In the fallopian tubes*          

12) How many eggs are typically released per cycle?     

            □ 1*    

            □ 2      

            □ 3      

            □ 4     

The following are likely to decrease a woman's chance of fertility:

                                                                         True                 False

13) Smoking…………………………………………….  □*                    □         

14) Occasional caffeine intake……………………….     □                      □*

15) Moderate alcohol consumption…………………..     □                      □*

16) Safely conducted pregnancy termination       …….... □                      □*

17) Obesity……………………………………………...   □*                    □         

18) Gonorrhea or chlamydia infection……………….     □*                    □

19) Prior use of oral contraceptive pills………………     □                      □*

20) Being underweight due to frequent exercise

or limited caloric intake……………………………         □*                    □

21) Using certain types of sexual lubricants………….     □*                    □

The remainder of the questions relate to infertility treatment:

22) In vitro fertilization (IVF) refers to an infertility treatment in which:

            □ A thin catheter is used to deposit a man's sperm past the cervix directly into the uterus

            □ A man's sperm and a woman's egg are combined inside a laboratory and the resulting embryo is transferred into the uterus*

            □ When a woman carries a pregnancy for another couple who cannot get pregnant

            □ Surgery is performed to harvest sperm from the man                                         

The following three questions refer to the most recent national statistics published by the Centers for Disease Control and Prevention and the Society for Assisted Reproductive Technology:

23) For a woman under 35 years old, undergoing IVF with her own eggs, what is the pregnancy rate per cycle?

            □ ≤5%             

            □ 6-20%                      

            □ 21-40%        

            □ 41-60%*      

            □ ≥60%          

24) For a woman over 44 years old, undergoing IVF with her own eggs, what is the pregnancy rate per cycle?

            □ ≤5%*

            □ 6-20%                      

            □ 21-40%        

            □ 41-60%        

            □ ≥60%          

 25) In women who are undergoing IVF, what is the percentage of pregnancies that result in twins?  

            □ ≤5%                         

            □ 6-20%*                    

            □ 21-35%        

            □ 36-45%        

26) What is the average cost of an IVF cycle in the United States?          

            □ $5,000                      

            □ $12,000*      

            □ $20,000        

            □ $50,000        

27) Intrauterine insemination (IUI) refers to a treatment in which:           

            □ A thin catheter is used to deposit a man's sperm past the cervix directly into the uterus*

            □ A man's sperm and a woman's egg are combined inside a laboratory and the resulting embryo is transferred into the uterus

            □ Sperm are deposited directly into the vagina ("turkey baster")

            □ Surgery is performed to harvest sperm from the man                           

28) Egg cryopreservation (freezing) refers to an infertility treatment in which:                 

            □ A single sperm is injected into an egg to preserve the egg's integrity

            □ A man's sperm and a woman's egg are combined inside a laboratory and then frozen

            □ Strips of ovarian tissue are surgically removed and frozen

            □ Eggs are frozen following ovarian stimulation and egg retrieval*

29) As per the largest published studies, when using frozen eggs from women less than 37 years old, what is the live birth rate per thawed egg?     

            □ ≤10%*                     

            □ 11-15%        

            □ 16-20%        

            □ 21-25% 

The asterisks (*) were added to indicate which data in the survey has a statistically significant p-value.      

Appendix B

**Table 4:** FIT-KS analysis and sub-analyses The asterisks (*) were added to indicate which data in the table has a statistically significant p-value. FIT-KS: Fertility and Infertility Treatment Knowledge Score; OB-GYN: obstetrics and gynecology; T/F: true/false; IVF: in vitro fertilization; IUI: intrauterine insemination

	Correct (%)	Incorrect (%)
At what age are women most fertile?	237 (81.4)	54 (18.6)
	No difference by stage of training (p=0.32)	
	No difference by age (p=0.83)	
	No difference by specialty (p=0.63)	
	No difference by parity (p=0.20)	
Over which age range does a woman's ability to get pregnant decline most precipitously?	172 (59.3)	118 (40.7)
	No difference by stage of training (p=0.20)	
	No difference by age (p=0.13)	
	No difference by specialty (p=0.24)	
	No difference by parity (p=0.071)	
Over the course of one month, what is the percent chance that a 30-year-old woman who is trying to get pregnant will get pregnant?	117 (40.3)	173 (59.7)
	No difference by stage of training (p=0.41)	
	30-39 years old more likely to answer correctly (p=0.025)*	
	No difference by specialty (p=0.85)	
	No difference by parity (p=0.65)	
Over the course of one month, what is the percent chance that a 40-year-old woman who is trying to get pregnant will get pregnant?	147 (50.9)	142 (49.1)
	No difference by stage of training (p=0.28)	
	No difference by age (p=0.58)	
	No difference by specialty (p=0.12)	
	No difference by parity (p=0.43)	
On average, for a woman in her peak reproductive years, what is the percent chance that a pregnancy will end in a miscarriage?	143 (49.3)	147 (50.7)
	No difference by stage of training (p=0.14)	
	No difference by age (p=0.53)	
	OB-GYN more likely to answer correctly (p=0.0027)*	
	No difference by parity (p=0.71)	
T/F: a woman and a man can both contribute to a couple's infertility	289 (99.3)	2 (0.7)
	No difference by stage of training (p=0.52)	
	No difference by age (p=0.71)	
	No difference by specialty (p=0.69)	
	No difference by parity (p=0.30)	
T/F: a man's age is a factor that affects a couple's fertility	203 (69.8)	88 (30.2)
	No difference by stage of training (p=0.12)	
	>40-year-olds more likely to answer correctly (p=0.028)*	
	No difference by specialty (p=0.85)	
	Parous more likely to answer correctly (p=0.014)*	
T/F: having less than nine periods in a year can be normal for some women and doesn't require any further evaluation	182 (62.5)	109 (37.5)
	No difference by stage of training (p=0.60)	
	No difference by age (p=0.27)	
	No difference by specialty (p=0.69)	
	No difference by parity (p=0.80)	
What is the average survival time of normal sperm in the female reproductive tract?	108 (37.2)	182 (62.8)
	No difference by stage of training (p=0.81)	
	No difference by age (p=0.34)	
	No difference by specialty (p=0.27)	
	No difference by parity (p=0.40)	
When is the optimal time to have sexual intercourse in order to get pregnant?	264 (91)	26 (9)
	Attendings more likely to answer correctly (p=0.0062)*	
	>40-year-olds more likely to answer correctly (p=0.048)*	
	No difference by specialty (p=0.084)	
	Parous more likely to answer correctly (p=0.014)*	
Where does fertilization most commonly occur?	238 (82.1)	52 (17.9)
	Medical students more likely to answer correctly (p=0.015)*	
	30-39-year-olds more likely to answer incorrectly (p=0.0025)*	
	No difference by specialty (p=0.097)	
	Nulliparous more likely to answer correctly (p=0.048)*	
How many eggs are typically released per cycle?	259 (89.9)	29 (10.1)
	Attendings least likely to answer correctly (p=0.035)*	
	26-29-year-olds more likely to answer correctly (p=0.024)*	
	No difference by specialty (p=0.11)	
	No difference by birth (p=0.37)	
T/F: smoking affects fertility	287 (99)	3 (1)
	No difference by stage of training (p=0.48)	
	No difference by age (p=0.37)	
	No difference by specialty (p=0.62)	
	No difference by parity (p=0.97)	
T/F: occasional caffeine intake affects fertility	260 (89.7)	30 (10.3)
	No difference by stage of training (p=0.85)	
	No difference by age (p=0.84)	
	No difference by specialty (p=0.11)	
	No difference by parity (p=0.69)	
T/F: moderate alcohol consumption affects fertility	125 (43.1)	165 (59.9)
	No difference by stage of training (p=0.091)	
	No difference by age (p=0.17)	
	No difference by specialty (0.077)	
	No difference by parity (p=0.24)	
T/F: safely conducted pregnancy termination affects fertility	234 (80.7)	56 (19.3)
	No difference by stage of training (p=0.71)	
	No difference by age (p=0.61)	
	No difference by specialty (p=0.091)	
	No difference by parity (p=0.37)	
T/F: obesity affects fertility	274 (94.5)	16 (5.5)
	No difference by stage of training (p=0.22)	
	No difference by age (p=0.89)	
	No difference by specialty (p=0.86)	
	No difference by parity (p=0.79)	
T/F: gonorrhea or chlamydia infection affects fertility	270 (93.1)	20 (6.9)
	Medical student least likely to answer correctly (p=0.038)*	
	No difference by age (p=0.12)	
	No difference by specialty (p=0.59)	
	No difference by parity (p=0.82)	
T/F: prior use of oral contraceptive pills affects fertility	248 (85.5)	42 (14.5)
	No difference by stage of training (p=0.44)	
	>40-year-olds least likely to answer correctly (p=0.020)*	
	No difference by specialty (p=0.051)	
	No difference by parity (p=0.50)	
T/F: being underweight due to frequent exercise or limited caloric intake affects fertility	283 (97.6)	7 (2.4)
	No difference by stage of training (p=0.98)	
	No difference by age (p=0.38)	
	OB-GYN more likely to answer incorrectly (0.0297)*	
	No difference by parity (p=0.74)	
T/F: using certain types of sexual lubricants affects fertility	171 (59)	119 (41)
	Attendings more likely to answer correctly (p=0.0013)*	
	>40-year-olds more likely to answer correctly; 26-29-year-olds more likely to answer incorrectly (p=0.0153)*	
	No difference by specialty (p=0.20)	
	Parous more likely to answer correctly (p=0.024)*	
IVF procedure definition	281 (97.2)	8 (2.8)
	No difference by stage of training (p=0.42)	
	No difference by age (p=0.52)	
	No difference by specialty (p=0.42)	
	No difference by parity (p=0.57)	
For a woman <35 years old undergoing IVF with her own eggs, what is the pregnancy rate per cycle?	58 (20.6)	224 (79.4)
	No difference by stage of training (p=0.80)	
	No difference by age (p=0.29)	
	No difference by specialty (p=0.64)	
	No difference by parity (p=0.46)	
For a woman over 44 years old, undergoing IVF with her own eggs, what is the pregnancy rate per cycle?	124 (44)	158 (56)
	No difference by stage of training (p=0.85)	
	No difference by age (p=0.48)	
	No difference by specialty (p=0.093)	
	No difference by parity (p=0.41)	
In women undergoing IVF, what is the percentage of pregnancies that result in twins?	124 (44)	158 (56)
	Medical students least likely to answer correctly (p=0.0057)*	
	<25-year-olds least likely to answer correctly (p=0.0054)*	
	No difference by specialty (p=0.88)	
	Parous more likely to answer correctly (p=0.0003)*	
Average cost of an IVF cycle in the United States	66 (23.4)	216 (76.6)
	No difference by stage of training (p=0.37)	
	No difference by age (p=0.099)	
	No difference by specialty (p=0.60)	
	No difference by birth (p=0.17)	
IUI treatment definition	261 (92.6)	21 (7.4)
	Attending physicians more likely to answer correctly (0.032)*	
	No difference by age (p=0.15)	
	No difference by specialty (p=0.20)	
	No difference by parity (p=0.47)	
Egg cryopreservation procedure definition	260 (92.2)	22 (7.8)
	No difference by stage of training (p=0.75)	
	No difference by age (p=0.54)	
	No difference by specialty (p=0.17)	
	No difference by parity (p=0.17)	
When using frozen eggs from women <37 years old, what is the live birth rate per thawed egg?	41 (14.5)	241 (85.5)
	Medical students least likely to answer correctly (p=0.047)*	
	No difference by age (p=0.061)	
	No difference by specialty (p=0.48)	
	Parous more likely to answer correctly (p=0.0039)*	
